# Compartmentalization of Intrarenal Programmed Cell Death Protein 1-Ligand 1 and Its Receptor in Kidney Injury Related to Immune Checkpoint Inhibitor Nephrotoxicity

**DOI:** 10.3389/fmed.2022.902256

**Published:** 2022-06-09

**Authors:** Désirée Tampe, Sarah Birgit Kopp, Eva Baier, Samy Hakroush, Björn Tampe

**Affiliations:** ^1^Department of Nephrology and Rheumatology, University Medical Center Göttingen, Göttingen, Germany; ^2^Institute of Pathology, University Medical Center Göttingen, Göttingen, Germany

**Keywords:** PD-L1, PD-1, checkpoint inhibition, kidney injury, CRP, complement system, complement C4

## Abstract

**Background:**

Due to advances in cancer therapy, immune checkpoint inhibitors (ICIs) are new classes of drugs targeting programmed cell death protein 1-ligand 1 (PD-L1) or its receptor (PD-1) used in many cancer therapies. Acute interstitial nephritis (AIN) is a potential and deleterious immune-related adverse events (irAE) and the most common biopsy-proven diagnosis in ICI-related nephrotoxicity. AIN in patients receiving ICIs is was only seen in cases with tubular PD-L1 positivity, while PD-1 expression is limited to inflammatory cells and also observed in injured kidneys independent of ICI therapy. We have previously described that PD-L1 positivity can also be detected in glomerular and endothelial compartments. We here aimed to describe compartmentalization of renal PD-L1 expression specifically in injured kidneys with confirmed nephrotoxicity related to ICIs, its association with presence of PD-1, and clinical findings.

**Methods:**

We included human kidney samples with AIN related to ICI therapy to describe PD-L1 and PD-1 expression localized to different renal compartments in association with clinical and laboratory parameters.

**Results:**

We herein report compartmentalization of PD-L1 with tubular positivity in all cases, partially overlapping with glomerular and endothelial PD-L1 positivity. Furthermore, we provide evidence that tubular PD-L1 in ICI-related nephrotoxicity correlates with levels of C-reactive protein (CRP), while glomerular and endothelial PD-L1 positivity with lower serum levels of complement component C4. Interestingly, glomerular PD-L1 correlated with kidney function, while interstitial cell PD-1 positivity specifically with severity of kidney injury. Finally, we provide evidence for signaling pathways associated with intrarenal PD-L1/PD-1 expression.

**Conclusion:**

Our findings implicate that that AIN related to ICI therapy requires presence of interstitial cells positive for PD-1, and that blocking PD-L1/PD-1 signaling may contribute to nephrotoxicity specifically related to these agents.

## Introduction

Due to recent advances in cancer therapy, immune checkpoint inhibitors (ICIs) are new classes of drugs targeting programmed cell death protein 1-ligand 1 (PD-L1, synonym CD274, B7) homolog 1) or its receptor (PD-1, synonym CD 279) (1). By blocking negative co-stimulatory pathways, ICIs allow T cells to remain activated and thereby enhance the anti-tumoral immune response. ICIs are now approved for the use in melanoma, small cell and non-small cell lung cancer, renal cell carcinoma and urothelial carcinoma, among others (1). However, despite benefits with respect to progression-free and overall cancer survival, upregulation of the immune system has also been associated with a wide spectrum of immune-related adverse events (irAE) in different organ systems (2). Among them, acute interstitial nephritis (AIN) is a potential and deleterious irAE in the kidney observed in patients receiving ICIs and is the most common biopsy-proven diagnosis in ICI-related nephrotoxicity (3, 4). AIN in patients receiving ICIs is was only seen in cases with tubular PD-L1 positivity, while PD-1 expression is limited to inflammatory cells and also observed in injured kidneys independent of ICI therapy (4). In addition to tubular PD-L1, we have previously described that PD-L1 positivity can also be detected in glomerular and endothelial compartments (5). Interestingly, PD-L1 is not present in control kidneys that might contribute to a specific involvement in injured kidneys and ICI-related nephrotoxicity (4, 5). In addition, these observations implicate distinct roles of PD-L1 among different renal compartments depending on its presence or absence. In the context of ICI-related nephrotoxicity, PD-L1 expression in distinct renal compartments has not yet been analyzed in nephrotoxicity related to ICI therapy thus far. Therefore, we here aimed to describe compartmentalization of renal PD-L1 expression in injured kidneys with confirmed nephrotoxicity related to ICIs, its association with presence of PD-1, and clinical findings.

## Methods

### Study Population

A total number of 7 cases with biopsy-proven AIN related to nephrotoxicity of ICI therapy were retrospectively included between 2015 and 2020 at the University Medical Center Göttingen, Germany. In parts, the patient cohort has previously been described (5). The studies involving human participants were reviewed and approved by the Institutional Review Board of the University Medical Center Göttingen, Germany (No. 22/2/14). The patients/participants provided their written informed consent for the use of routinely collected data for research purposes as part of their regular medical care in the contract of the University Medical Center Göttingen. Medical records were used to obtain data on age, sex, medication, date of biopsy, laboratory results and urinary analysis. The estimated glomerular filtration rate (eGFR) was calculated using the Chronic Kidney Disease Epidemiology Collaboration (CKD-EPI) equation (6).

### Renal Histopathology and Immunohistochemistry

A renal pathologist evaluated the kidney biopsies and was blinded to clinical data. Formalin-fixed, paraffin-embedded kidney sections were deparaffinized in xylene and rehydrated in ethanol containing distilled water. Tissue sections were stained using primary antibodies against PD-L1 (1:100, ab205921, Abcam, Cambridge, United Kingdom), PD-1 (1:500, ab52587, Abcam, Cambridge, United Kingdom), C1q (1:30,000, A0136, Agilent Dako, Santa Clara, United States), and C3c (1:10,000, A0062, Agilent Dako, Santa Clara, United States), labeling was performed using Novolink^TM^ Polymer Detection System (Leica Biosystems, Wetzlar, Germany) according to the manufacturer's protocol. Nuclear counterstain was performed by using Mayer's Hematoxylin Solution (Sigma, St. Louis, United States). Kidney biopsies were evaluated for presence/absence of PD-L1 and PD-1 deposits in the glomerular tuft, interlobular arteries, peritubular capillaries, and venules. For quantitative analysis, the intensity of PD-L1 staining was evaluated at 400 × magnification and scored semiquantitatively (0: no staining, 1: weak and segmental staining, 2: moderate staining, 3: strong staining). Interstitial cells positive for PD-1 were evaluated by using mean values of 10 randomly selected cortical visual fields at 400 × magnification and scored semiquantitative (0: no cell per visual field, 1: 1–3 cells per visual field, 2: 3–6 cells per visual field, 3: >6 cells per visual field).

### Plasma C3c and C4 Measurements

Plasma concentrations of human complement components C3c (9D9621, Abbott, Chicago, United States) and C4 (9D9721, Abbott, Chicago, United States) were determined by turbidimetric measurements on the ARCHITECT-C module. Normal range plasma concentrations for circulating C3c are defined between 0.82–1.93 g/L, and C4 between 0.15–0.57 g/L.

### Analyses of Publicly Available Array Datasets

Publicly available datasets for PD-L1 expression (encoded by *CD274*) from Nephroseq (www.nephroseq.org, May 2022, University of Michigan, Ann Arbor, MI) were analyzed according to general recommendations (7, 8). Particularly, median-centered log_2_ *CD274* mRNA expression levels (reporter ID: 29126, platform: Affymetrix Human Genome U133 Plus 2.0 Array, altCDF v10) were extracted specifically from microdissected tubulointerstitial compartments (9). Pathway analysis was performed for gene enrichment associated with tubulointerstitial *CD274* expression with a correlation threshold of ≥0.2 by using reactome (http://reactome.org), pathways with a predefined entities value of *p* ≤ 0.01 were included and shown in Supplementary Tables 1, 2 (10).

### Statistical Methods

Variables were tested for normal distribution using the Shapiro-Wilk test. Statistical comparisons were not formally powered or pre-specified. Continuous and ordinal variables were presented as mean ± standard deviation, categorical variables as percentages of total. Spearman's correlation was performed to assess correlations and heatmaps reflect the mean values of Spearman's ρ. A Spearman's ρ more than ±0.6 in the correlation matrix was defined as relevant indicated by rectangle boxes, and independent statistical evaluation of these parameters was performed by linear regression. A probability (*p*) value of <0.05 was considered statistically significant. Data analyses were performed with GraphPad Prism (version 8.4.3 for MacOS, GraphPad Software, San Diego, CA, United States), linear regression analyses were performed using IBM SPSS Statistics (version 27 for MacOS, IBM Corporation, Armonk, NY, United States).

## Results

### PD-L1 Localizes to Different Compartments in ICI-Related Nephrotoxicity

**Figure 1:**
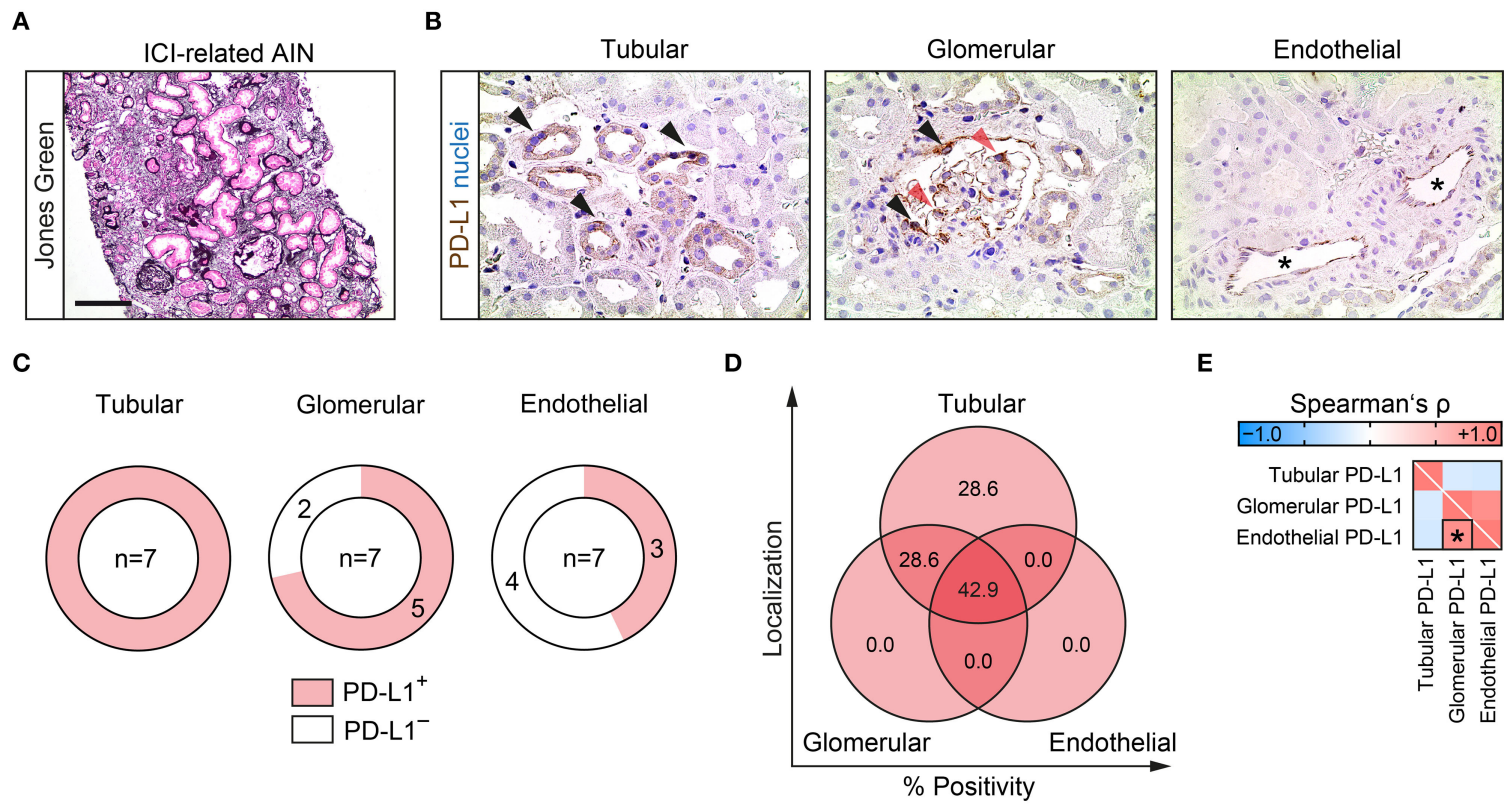
PD-L1 localizes to different compartments in ICI-related nephrotoxicity. **(A)** Representative kidney biopsy with AIN related to ICI therapy stained for Jones Green (scale bar: 200 μm). **(B)** PD-L1 positivity within tubular (proximal tubular epithelial cells depicted by black arrowheads), glomerular (parietal epithelial cells depicted by black arrowheads, and podocytes by red arrowheads) and endothelial compartments (asterisks). **(C,D)** Frequency of PD-L1 positivity among different renal compartments. **(E)** Correlative analysis of PD-L1 positivity within different renal compartments in ICI-related nephrotoxicity are shown by heatmap reflecting mean values of Spearman's ρ. The rectangle box indicates a Spearman's ρ more than ±0.6, the asterisk a significant correlation in the linear regression analysis (*p* < 0.05). AIN, acute interstitial nephritis; ICI, immune checkpoint inhibitor; PD-L1, programmed cell death protein 1-ligand 1.

We included a total number of 7 kidney specimens with ICI-related nephrotoxicity (6 cases with ICI therapy targeting PD-1 and 1 targeting PD-L1, Figure 1A, Table 1). PD-L1 expression localized to different renal compartments was systematically analyzed, including tubular, glomerular and endothelial PD-L1 positivity. Tubular PD-L1 positivity was predominantly found in proximal tubular epithelial cells, glomerular PD-L1 was found in parietal epithelial cells and podocytes (Figure 1B). Tubular PD-L1 positivity was present in all cases of ICI-related nephrotoxicity. Moreover, glomerular PD-L1 positivity was only found in 5/7 (71.4%) and endothelial PD-L1 positivity in 3/7 (42.9%) cases (Figure 1C). Comparison of PD-L1 abundance within distinct compartments revealed that PD-L1 was present in all renal compartments in 3/7 (42.9%), followed by only tubular and tubular/glomerular PD-L1 positivity in 2/7 (28.6%), respectively (Figure 1D). In contrast, we did not detect isolated glomerular or endothelial PD-L1 positivity in injured kidneys with ICI-related nephrotoxicity (Figure 1D). Correlative analysis revealed that the only significant association was observed for glomerular and endothelial PD-L1 positivity in ICI-related nephrotoxicity (Figure 1E). In summary, PD-L1 was present in distinct renal compartment in nephrotoxicity related to ICI therapy.

**Table 1:** Clinical, laboratory and histopathological parameters of the total cohort.

**Clinical data**	**Value**	
Female sex—no. (%)	2 (28.8)	
Age—years	72.6 ± 7.1	
PD-1 targeted therapy—no. (%)	6 (85.7)	
PD-L1 targeted therapy—no. (%)	1 (14.3)	
**Laboratory parameters**	**Value**	**Normal range**
Hemoglobin—g/dL	10.5 ± 1.8	13.5–17.5
Thrombocytes— ×10^3^/μL	290.1 ± 83.6	150–350
Leukocytes— ×10^3^/μL	9 ± 2.8	4–11
TSH—mIU/L	0.8 ± 0.7	0.35–4.94
Sodium—mmol/L	139.7 ± 2.9	136–145
Potassium—mmol/L	4.2 ± 0.6	3.5–4.6
Calcium—mmol/L	2.2 ± 0.2	2.2–2.55
Serum creatinine—mg/dL	3.4 ± 1.6	0.7–1.2
eGFR—mL/min	21.7 ± 11.2	> 60
BUN—mg/dL	52.6 ± 20.7	8–26
CRP—mg/L	44.7 ± 39.1	≤ 5
LDH—U/L	290.4 ± 78.6	125–250
Complement C3c—g/L	1.2 ± 0.2	0.82–1.93
Complement C4—g/L	0.3 ± 0.1	0.15–0.57
**Urinary parameters**	**Value**	**Normal range**
Proteinuria—mg/L	394.4 ± 523.5	<140
Albuminuria/g creatinine—mg/g	182.4 ± 232	<30
Urinary α_1_-microglobulin—mg/L	47.2 ± 29	<12
Urinary IgG—mg/L	32 ± 49.2	<9.6
Urinary kappa—mg/L	51.7 ± 51.4	<6.8
Urinary lambda—mg/L	22.4 ± 32.8	<3.7
Urinary kappa/lambda—ratio	3.5 ± 1.9	>1 or <5.2
**Histopathological findings**	**Value**	
Glomerular sclerosis—% of total	10.9 ± 11	
IF/TA—%	35 ± 22.5	
C1q positivity—no. (%)	0 (0)	
C3c positivity—no. (%)	0 (0)	

*AIN, acute interstitial nephritis; BUN, blood urea nitrogen; C1q, complement factor 1q; C3c, complement factor 3 conversion product; C4, complement factor 4; CRP, C-reactive protein; eGFR, estimated glomerular filtration rate (CKD-EPI); IF/TA, interstitial fibrosis/tubular atrophy; IgG, immunoglobulin G; LDH, lactate dehydrogenase; no., number; PD-L1, programmed cell death protein 1-ligand 1; TSH, thyroid stimulating hormone*.

### Compartmentalization of Renal PD-L1 Is Associated With Systemic Inflammation and Complement System Activation

**Figure 2:**
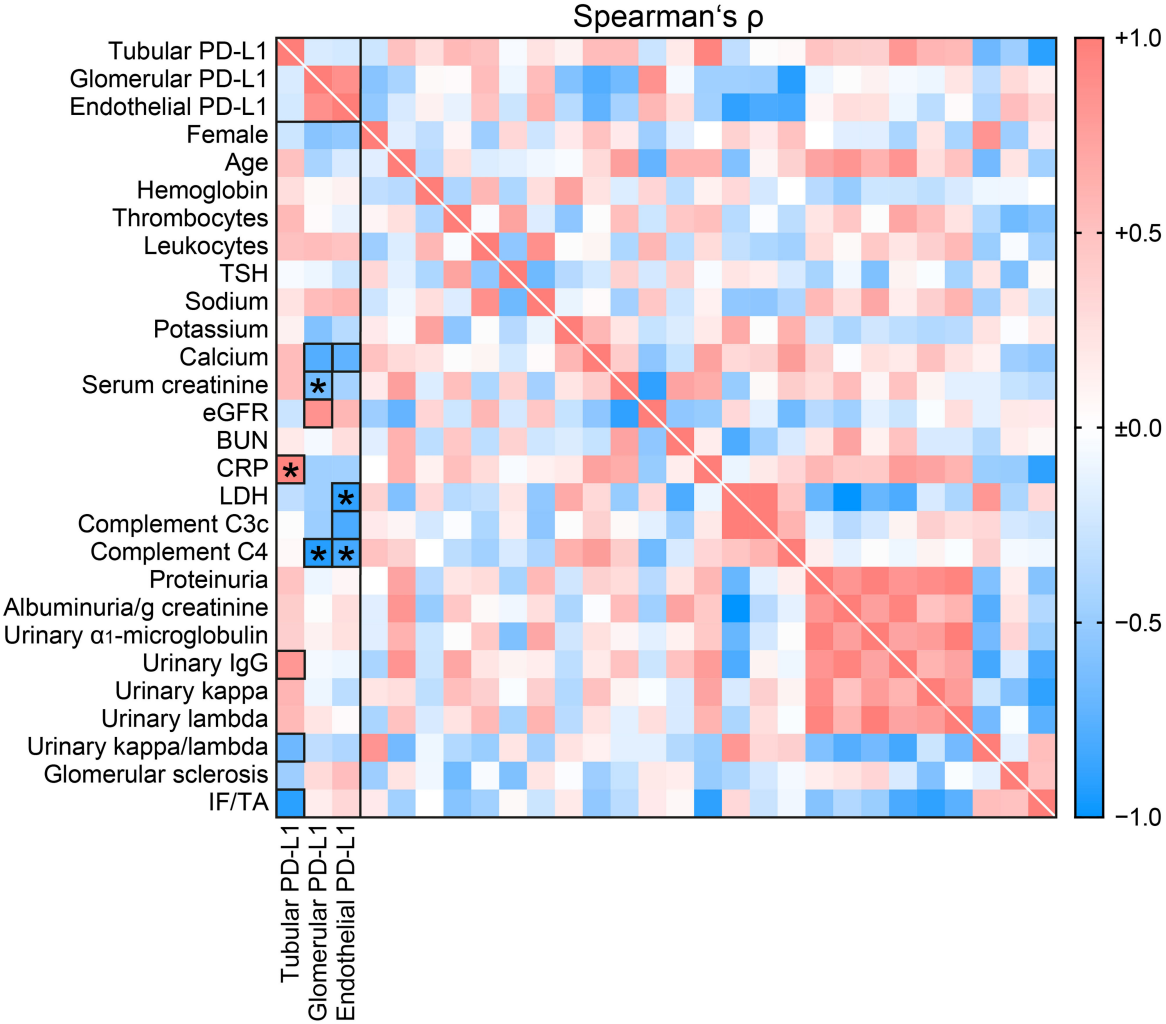
Compartmentalization of renal PD-L1 is associated with systemic inflammation and complement system activation. Intensity of PD-L1 positivity within different renal compartments in ICI-related nephrotoxicity in association with clinical and laboratory parameters are shown by heatmap reflecting mean values of Spearman's ρ. Rectangle boxes indicate a Spearman's ρ more than ±0.6, asterisks significant correlations in the stepwise linear regression analysis (*p* < 0.05). AIN, acute interstitial nephritis; BUN, blood urea nitrogen; C3c, complement factor 3 conversion product; C4, complement factor 4; CRP, C-reactive protein; eGFR, estimated glomerular filtration rate (CKD-EPI); ICI, immune checkpoint inhibitor; IF/TA, interstitial fibrosis/tubular atrophy; IgG, immunoglobulin G; LDH, lactate dehydrogenase; PD-L1, programmed cell death protein 1-ligand 1; TSH, thyroid stimulating hormone.

To gain insights into potential factors affecting PD-L1 compartmentalization in nephrotoxicity related to ICI therapy, we next compared clinical and laboratory parameters with PD-L1 localized to different renal compartments (Table 1). Intensity of tubular PD-L1 positivity correlated with serum levels of C-reactive protein (CRP, *p* = 0.001, Figure 2, Table 2). Interestingly, no such association was observed for glomerular and endothelial PD-L1 (Figure 2), implicating a specific involvement of systemic inflammation in PD-L1 abundance within the tubular compartment in nephropathy related to ICI therapy. Moreover, glomerular PD-L1 inversely correlated with serum levels of creatinine (*p* = 0.0232, Figure 2, Table 2) and lower levels of complement C4 in ICI-related nephrotoxicity (*p* = 0.0033, Figure 2, Table 2), independent of hypocomplementemia *per se* or intrarenal complement deposits (Table 1). A comparable association with complement system activation was also observed for endothelial PD-L1 correlated with low serum levels of complement C4 (*p* = 0.0313, Figure 2, Table 2) and lactate dehydrogenase (LDH, *p* = 0.0194, Figure 2, Table 2). In summary, compartmentalization of renal PD-L1 is associated with systemic inflammation and complement system activation.

**Table 2:** Linear regression analyses of PD-L1.

**Comparison with tubular PD-L1**	**β**	***p* value**
CRP—mg/L	0.9741	0.0010
Urinary IgG—mg/L	0.1858	0.1823
Urinary kappa/lambda—ratio	−0.1646	0.1970
IF/TA—%	0.0038	0.9920
**Comparison with glomerular PD-L1**	**β**	**p value**
Calcium—mmol/L	−0.2022	0.0885
Creatinine—mg/dL	−0.4099	0.0242
eGFR—mL/min	−0.1327	0.6945
Complement C4—g/L	−0.7281	0.0033
**Comparison with endothelial PD-L1**	**β**	**p value**
Calcium—mmol/L	−0.0270	0.8489
LDH—U/L	−0.6222	0.0194
Complement C3c—g/L	−0.1741	0.3789
Complement C4—g/L	−0.4849	0.0313

*C3c, complement factor 3 conversion product; C4, complement factor 4; CRP, C-reactive protein; eGFR, estimated glomerular filtration rate (CKD-EPI); IF/TA, interstitial fibrosis/tubular atrophy; IgG, immunoglobulin G; LDH, lactate dehydrogenase; PD-L1, programmed cell death protein 1-ligand 1*.

### PD-1 Is Present in Interstitial Cells and Associates With Kidney Injury in ICI-Related Nephrotoxicity

**Figure 3:**
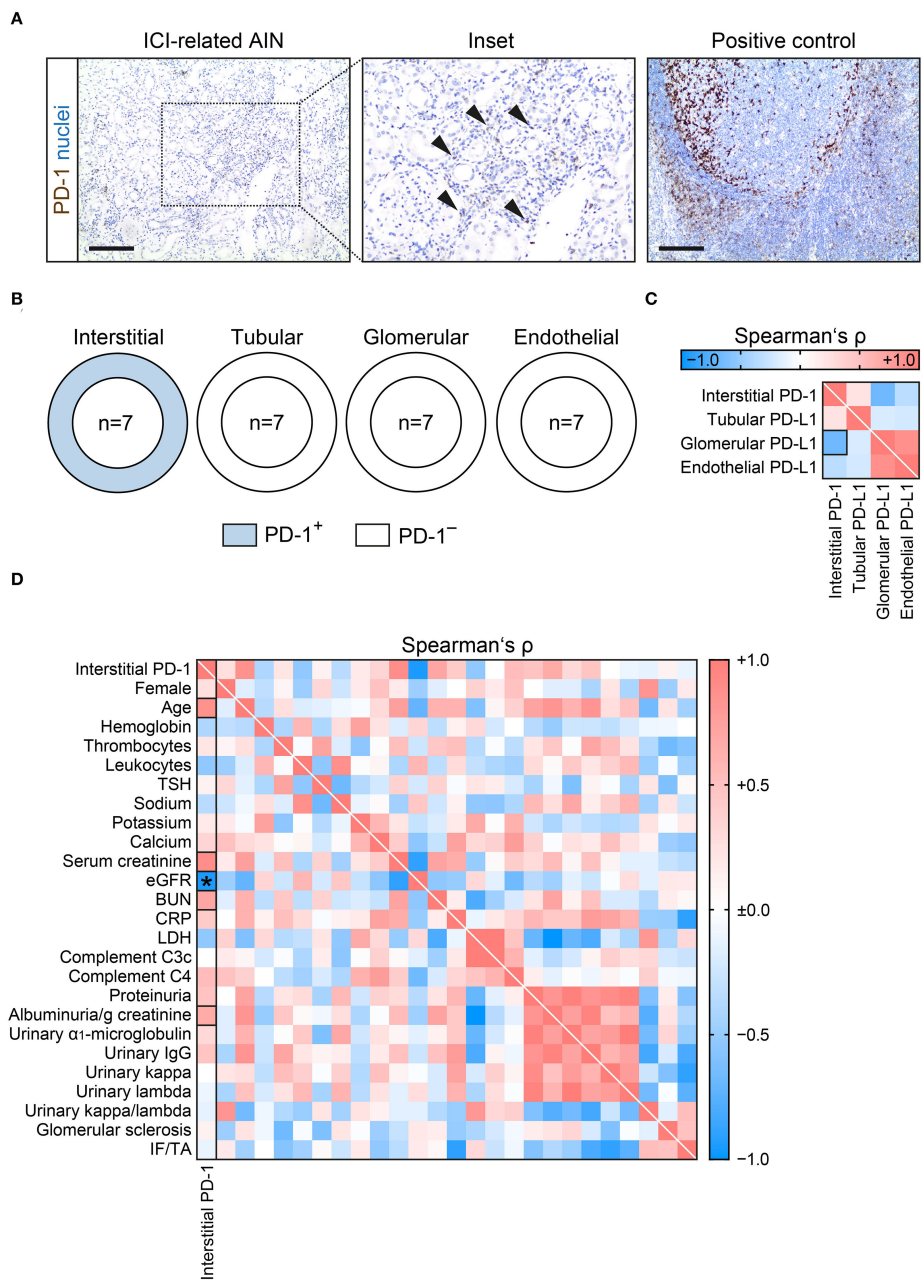
PD-1 is present in interstitial cells and associates with kidney injury in ICI-related nephrotoxicity. **(A)** Interstitial cell PD-1 positivity (arrowheads) in AIN related to ICI therapy, tonsil tissue was used as positive control (scale bar: 200 μm). **(B)** Frequency of PD-1 positivity among different renal compartments. **(C)** Correlative analysis of interstitial PD-1 and PD-L1 positivity within different renal compartments in ICI-related nephrotoxicity are shown by heatmap reflecting mean values of Spearman's ρ. The rectangle box indicates a Spearman's ρ more than ±0.6. **(D)** Extent of interstitial cells positive for PD-1 in ICI-related nephrotoxicity in association with clinical and laboratory parameters are shown by heatmap reflecting mean values of Spearman's ρ. Rectangle boxes indicate a Spearman's ρ more than ±0.6, asterisks significant correlations in the stepwise linear regression analysis (*p* < 0.05). AIN, acute interstitial nephritis; BUN, blood urea nitrogen; C3c, complement factor 3 conversion product; C4, complement factor 4; CRP, C-reactive protein; eGFR, estimated glomerular filtration rate (CKD-EPI); ICI, immune checkpoint inhibitor; IF/TA, interstitial fibrosis/tubular atrophy; IgG, immunoglobulin G; LDH, lactate dehydrogenase; PD-1, programmed cell death protein 1; PD-L1, programmed cell death protein 1-ligand 1; TSH, thyroid stimulating hormone.

Because previous studies implicated that susceptibility to develop renal complications related to ICI therapy requires presence of PD-1 and PD-L1, we next evaluated presence of PD-1 (4). PD-1 was present in interstitial cells and absent in the other intrarenal compartments (Figures 3A,B), confirming previous reports (4). There was no significant association between interstitial PD-1 and PD-L1 positivity in the tubular, glomerular or endothelial compartment (Figure 3C). Extent of interstitial cells positive for PD-1 correlated specifically with severity of kidney injury reflected by eGFR loss (*p* = 0.0016, Figure 3D, Table 3). In summary, presence of interstitial cells positive for PD-1 associated with kidney injury in nephrotoxicity related to ICI therapy.

**Table 3:** Linear regression analyses of PD-1.

**Comparison with interstitial PD-1**	**β**	***p* value**
Age—years	0.2999	0.1243
Creatinine—mg/dL	−0.2893	0.4450
eGFR—mL/min	−0.0759	0.0016
BUN—mg/dL	0.2082	0.3026
Albuminuria/g creatinine—mg/g	0.0952	0.5936

*BUN, blood urea nitrogen; eGFR, estimated glomerular filtration rate (CKD-EPI); PD-1, programmed cell death protein 1*.

### Identification of Intrarenal Signaling Pathways Associated With Tubulointerstitial PD-L1/PD-1

**Figure 4:**
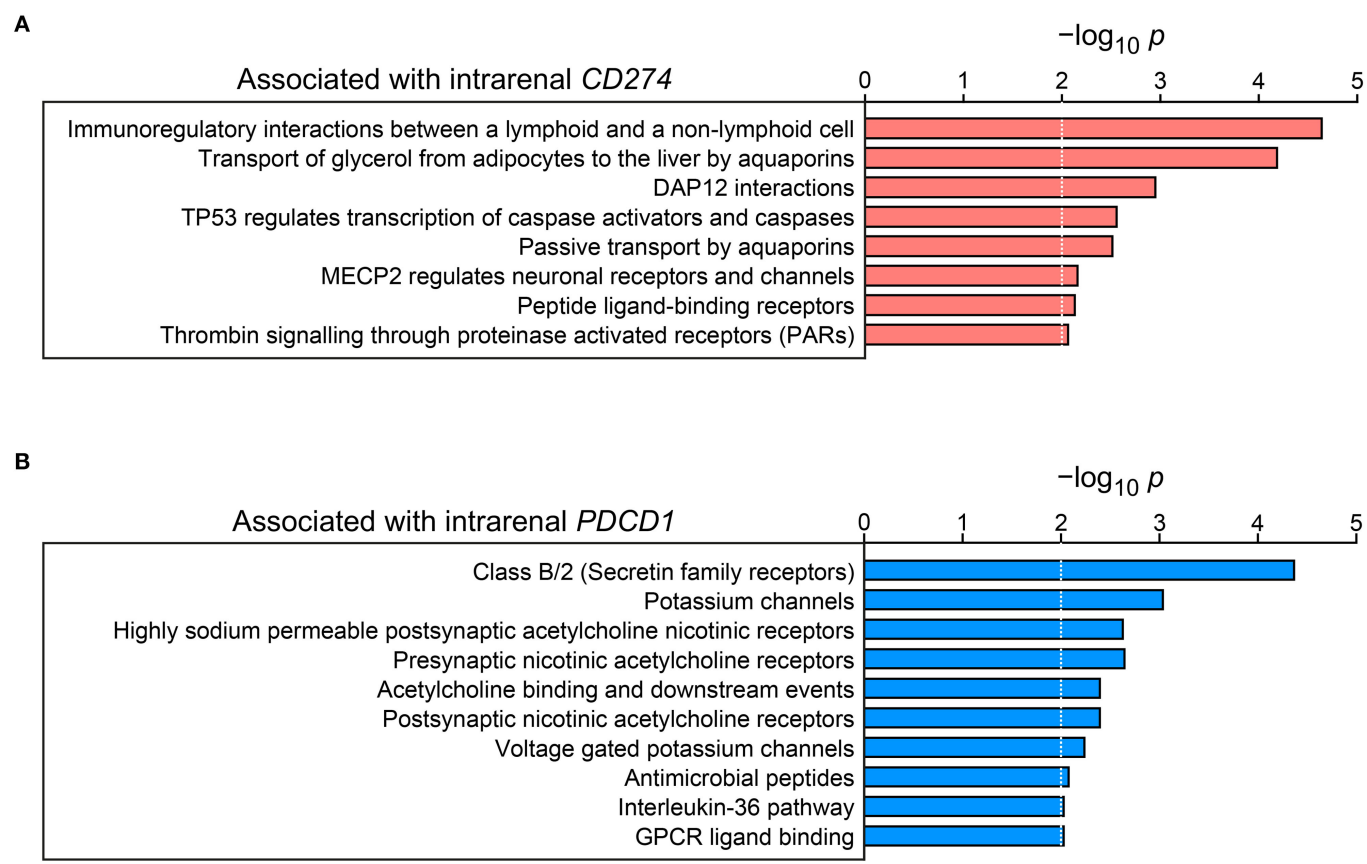
Identification of signaling pathways associated with tubulointerstitial PD-L1 and PD-1. **(A)** Entities –log_10_ *p* values of signaling pathways for gene enrichment positively associated with tubulointerstitial *CD274* mRNA expression are shown (the dotted lines correspond to the predefined threshold value of *p* ≤ 0.01). **(B)** Entities –log_10_ *p* values of signaling pathways for gene enrichment positively associated with tubulointerstitial *PDCD1* mRNA expression are shown (the dotted lines correspond to the predefined threshold value of *p* ≤ 0.01). PD-1, programmed cell death protein 1; PD-L1, programmed cell death protein 1-ligand 1.

To gain insights into intrarenal signaling pathways correlating with PD-L1/PD-1, we finally extracted transcriptome datasets for PD-L1 expression (encoded by *CD274*) and PD-1 (encoded by *PDCD1*) specifically from microdissected tubulointerstitial compartments (www.nephroseq.org, May 2022, University of Michigan, Ann Arbor, MI) (9). Gene set enrichment analysis linking *CD274* expression to potential signaling pathways revealed positive associations with immunoregulation and p53/caspase regulation (Figure 4A; Supplementary Table 1). Contrasting to this, tubulointerstitial *PDCD1* correlated with secretin family receptors, potassium and acetylcholine signaling, and interleukin-36 (IL-36) pathway molecules (Figure 4B; Supplementary Table 2). In summary, tubulointerstitial PD-L1 and PD-1 correlated with distinct signaling pathways in the kidney that might contribute to nephrotoxicity related to ICI therapy.

## Discussion

We here expand the current knowledge of renal PD-L1 compartmentalization specifically in nephrotoxicity related to ICI therapy. AIN is a deleterious irAE in the kidney and the most common histopathologic correlate of nephrotoxicity related to ICI therapy (4). On a mechanistic level, previous studies suggested that AIN related to ICIs is associated with tubular upregulation of PD-L1 reflecting susceptibility to develop irAE (4). *In vitro* experiments revealed that interferon-gamma-(INF-γ) stimulated PD-L1-positive tubular epithelial cells function as “non-professional” antigen presenting cells (APCs) activating co-inhibitory PD-1 signaling on cytotoxic CD8-positive T cells resulting in T cell anergy, apoptosis and exhaustion, thus contributing to mechanisms of so-called immune evasion (11). In addition to tubular PD-L1, we here described that PD-L1 positivity can also be detected in glomerular and endothelial compartments. These observations reveal distinct roles of PD-L1 among different renal compartments. As described previously, all cases of ICI-related nephrotoxicity featured PD-L1 positivity (4, 5). Regarding the distinct compartmentalization of PD-L1 expression, PD-L1 positive immunostaining occurred in most cases in all three compartments, followed by either tubular or combined tubular and glomerular PD-L1 positivity. Interestingly, we did neither detect isolated glomerular nor endothelial PD-L1 positivity. Tubular PD-L1 positivity was predominantly found in proximal tubular epithelial cells as previously described (12, 13). Glomerular PD-L1 was found in parietal epithelial cells and podocytes, again in line with previous reports (14). Among potential mechanisms, tubular PD-L1 positivity was positively correlated with systemic inflammation (elevated serum levels of CRP), in line with previous investigations reporting CRP serum elevation to reflect irAE onset including kidney involvement (15, 16). Interestingly, this association was exclusively restricted to the tubular compartment hinting at an involvement of systemic inflammation in the setting of AIN in ICI-related nephrotoxicity. Elevated levels of CRP have also been described in AIN related to other drugs, limiting its value as biomarker especially in cancer patients where systemic inflammation is frequently observed and influenced by tumor burden (17, 18). Contrasting to this, glomerular and endothelial PD-L1 expression featured no such association with elevated serum levels of CRP. Glomerular PD-L1 correlated with kidney function and has previously been connected with protection from injury in experimental models of glomerular injury (19–22). Moreover, glomerular and endothelial PD-L1 correlated with lower serum levels of C4 in ICI-related nephrotoxicity, independent of hypocomplementemia *per se* or intrarenal complement deposits. These findings implicate that the complement system might regulate intrarenal PD-L1 expression independent of injury directly attributed to complement deposits in the kidney. The versatile functionality of the complement system has gained increasing interest in the context of PD-L1/PD-1 signaling, especially in the field of cancer research, where therapy non-responding to PD-L1/PD-1-targeting agents poses an ongoing challenge, despite resounding treatment success in many solid tumor entities particularly of advanced stages (23). Representing a hierarchically structured part of the innate immunity, cell surface-bound or intracellular proteins are known to engage in a self-amplifying complement cascade resulting in the assembly of the so-called membrane attack complex (MAC), also known as C5b-9 (24). Modes of activation comprise the classical, the alternative or the lectin pathway, wherein immune modulatory functions of the complement system contribute equally to balance immune homeostasis. Amongst others complement inhibiting factor H triggers the production of a tolerogenic cytokine profile, thereby allowing immune evasion and damage control. Under certain conditions, endothelial cells feature the ability to alter their surface expression profile for adhesion molecules thus preventing cytotoxic T cell infiltration (23). In the tumor microenvironment, T-cell trafficking, and tissue infiltration of CD8-positive T cells via endothelial cells require complement activation, local production of C3 and C5a (23). Interestingly, complement components have already been described to regulate PD-L1 expression and signaling. Particularly, complement component C5a has been shown to induce PD-L1 on monocytes (25). In addition, deletion of C3 in cancer cells has been shown to enhance the efficacy of anti-PD-L1 treatment (26). Most recently, increased PD-L1 has also been associated with complement system activation in patients with paroxysmal nocturnal hemoglobinuria (PNH) (27). Based on our observations, an interplay between PD-L1 and complement system activation may also be crucial in the pathogenesis of nephrotoxicity related to ICI therapy. Behind the background of the PD-L1-positive glomerular and endothelial compartments exposing the highest degree of complement involvement, while tubular PD-L1 positivity correlated with systemic inflammation, distinct modes of damage mediation attenuating irAE-related T cell cytotoxicity depending on the respective renal compartment might be reflected here. We here provide a direct link between PD-L1 signaling and complement involvement in the context of ICI-related nephrotoxicity independent of kidney injury directly attributed to intrarenal complement deposits.

Regarding PD-1, we here confirm previous reports that PD-1 positivity in AIN related to ICI therapy is limited to interstitial inflammatory cells (4). Intrarenal positivity for PD-1 has been described to be non-specific, as it was seen in inflammatory cells irrespective of the AIN etiology (4). Observed association between PD-1 positivity with severity of kidney injury implicate that blocking PD-L1/PD-1 signaling may contribute to nephrotoxicity specifically related to ICI therapy. Interestingly, tubulointerstitial PD-L1 and PD-1 correlated with distinct signaling pathways in the kidney that might contribute to nephrotoxicity related to ICI therapy. Therefore, identification of mechanisms regulating intrarenal PD-L1/PD-1 (including the complement system) may provide an attractive therapeutical target to modulate susceptibility or treat to develop nephrotoxicity related to ICI therapy. This requires further investigation regarding its clinical implication.

This study has several limitations due to its retrospective design, the small patient number, and no independent validation. However, nephrotoxicity related to ICI therapy is a rare event with only a limited number of kidney biopsies (28–30). Our observations that PD-L1/PD-1 was present within distinct renal compartments associated with distinct systemic inflammatory markers and kidney injury might contribute to mechanistic insights into ICI-related nephrotoxicity.

## Data Availability Statement

The original contributions presented in the study are included in the article/Supplementary Material, further inquiries can be directed to the corresponding authors.

## Ethics Statement

The studies involving human participants were reviewed and approved by the Ethics Committee of the University Medical Center Göttingen. The patients/participants provided their written informed consent to participate in this study.

## Author Contributions

BT conceived the study, collected and analyzed data, and wrote the first draft. DT, SBK, EB, and SH collected and analyzed data. All authors contributed to the article and approved the submitted version.

## Funding

BT was supported by the Research Program, University Medical Center Göttingen (1403720). EB was funded by the Else-Kröner Research Program entitled Molecular Therapy and Prediction of Gastrointestinal Malignancies (7-67-1840876). We also acknowledge support by the Open Access Publication Funds of the Göttingen University. The funding sources were not involved in the design, collection, analysis, interpretation, writing or decision to submit the article.

## Conflict of Interest

The authors declare that the research was conducted in the absence of any commercial or financial relationships that could be construed as a potential conflict of interest.

## Publisher's Note

All claims expressed in this article are solely those of the authors and do not necessarily represent those of their affiliated organizations, or those of the publisher, the editors and the reviewers. Any product that may be evaluated in this article, or claim that may be made by its manufacturer, is not guaranteed or endorsed by the publisher.
