# Supplementary material for: Compartmentalization of Intrarenal Programmed Cell Death Protein 1-Ligand 1 and Its Receptor in Kidney Injury Related to Immune Checkpoint Inhibitor Nephrotoxicity
Source: Front Med (Lausanne). 2022 Jun 9;9:902256. doi: 10.3389/fmed.2022.902256 (PMC9218249; doi:10.3389/fmed.2022.902256)
Supplement: Supplementary file 1 [file Data_Sheet_1.pdf]

Compartmentalization of intrarenal programmed cell death protein 1-ligand 1 and its receptor in kidney injury related to immune checkpoint inhibitor nephrotoxicity

Supplementary Material

Désirée Tampe[1], Sarah Birgit Kopp[1], Eva Baier[1], Samy Hakroush[2], Björn Tampe[1,#]

[1]*Department of Nephrology and Rheumatology, University Medical Center Göttingen, Göttingen, Germany*

[2]*Institute of Pathology, University Medical Center Göttingen, Göttingen, Germany*

[#]*Corresponding author*

Running title: Renal PD-L1/PD-1 in ICI-related nephrotoxicity

Corresponding author:

Björn Tampe, MD

Department of Nephrology and Rheumatology

University Medical Center Göttingen

Georg August University

Göttingen, Germany

Email: bjoern.tampe@med.uni-goettingen.de

Supplementary Table 1. Pathways correlated with tubulointerstitial *CD274* mRNA expression.

Pathway	Entities found	Entities total	Entities ratio	Entities p	Entities FDR	Species
Immunoregulatory interactions between a Lymphoid and a non-Lymphoid cell	8	316	0.021	*2.13E-5*	*4.3E-3*	Homo sapiens
Transport of glycerol from adipocytes to the liver by Aquaporins	2	3	0	*6.03E-5*	*6.09E-3*	Homo sapiens
DAP12 interactions	3	53	0.004	*1.04E-3*	*6.98E-2*	Homo sapiens
TP53 Regulates Transcription of Caspase Activators and Caspases	2	20	0.001	*2.57E-3*	*1.13E-1*	Homo sapiens
Passive transport by Aquaporins	2	21	0.001	*2.83E-3*	*1.13E-1*	Homo sapiens
MECP2 regulates neuronal receptors and channels	2	32	0.002	*6.41E-3*	*1.93E-1*	Homo sapiens
Peptide ligand-binding receptors	4	203	0.013	*6.88E-3*	*1.93E-1*	Homo sapiens
Thrombin signalling through proteinase activated receptors (PARs)	2	36	0.002	*8.03E-3*	*2.01E-1*	Homo sapiens

Supplementary Table 2. Pathways correlated with tubulointerstitial *PDCD1* mRNA expression.

Pathway	Entities found	Entities total	Entities ratio	Entities p	Entities FDR	Species
Class B/2 (Secretin family receptors)	18	99	0.007	*4,00E-05*	*4.32E-2*	Homo sapiens
Potassium Channels	16	107	0.007	*8.48E-4*	*4.67E-1*	Homo sapiens
Highly sodium permeable postsynaptic acetylcholine nicotinic receptors	4	9	0.001	*2.19E-3*	*4.83E-1*	Homo sapiens
Presynaptic nicotinic acetylcholine receptors	5	15	0.001	*2.2E-3*	*4.83E-1*	Homo sapiens
Acetylcholine binding and downstream events	5	17	0.001	*3.73E-3*	*5.86E-1*	Homo sapiens
Postsynaptic nicotinic acetylcholine receptors	5	17	0.001	*3.73E-3*	*5.86E-1*	Homo sapiens
Voltage gated Potassium channels	8	44	0.003	*5.31E-3*	*7.27E-1*	Homo sapiens
Antimicrobial peptides	15	123	0.008	*7.75E-3*	*8.13E-1*	Homo sapiens
GPCR ligand binding	51	606	0.04	*8.69E-3*	*8.13E-1*	Homo sapiens
Interleukin-36 pathway	3	7	0	*8.7E-3*	*8.13E-1*	Homo sapiens
